# Medication Safety Gaps in Older Adults Across Outpatient and Inpatient Care in Peru: Evidence from Beers and STOPP/START Criteria

**DOI:** 10.3390/healthcare14152314

**Published:** 2026-07-31

**Authors:** Geraldine Y. Canaza-Chambi, Miguel A. Arce-Huamani

**Affiliations:** Academic Program of Human Medicine, Faculty of Health Sciences, Universidad Privada Norbert Wiener, Lima 15046, Peru; a2017100122@uwiener.edu.pe

**Keywords:** medication safety, older adults, potentially inappropriate prescribing, prescribing omissions, Beers Criteria, STOPP/START criteria

## Abstract

**Background/Objectives**: This study compared medication safety gaps across outpatient and inpatient care among older adults using explicit prescribing-quality criteria. **Methods**: We conducted an analytical cross-sectional study based on a retrospective review of 157 medical records from adults aged ≥ 60 years treated at Hospital Carlos Monge Medrano, a public referral hospital in Juliaca, Peru, in 2024. Stratified probability sampling included 73 outpatient and 84 inpatient records. Primary outcomes were Beers-defined potentially inappropriate medications, Screening Tool of Older Persons’ Prescriptions (STOPP)-defined potentially inappropriate prescribing, and Screening Tool to Alert to Right Treatment (START)-defined potential prescribing omissions. Overall medication safety gaps were assessed as a secondary exploratory composite outcome. Crude and adjusted prevalence ratios (PRs) with 95% confidence intervals (CIs) were estimated using modified Poisson regression with robust variance. **Results**: Overall medication safety gaps were identified in 79.0% of participants. START-defined omissions occurred in 52.9%, STOPP-defined inappropriate prescribing in 49.7%, and Beers-defined potentially inappropriate medications in 46.5%. Compared with outpatient care, hospitalization was associated with a lower prevalence of STOPP-defined inappropriate prescribing (adjusted PR = 0.74; 95% CI: 0.55–0.99) and a higher prevalence of START-defined omissions (adjusted PR = 1.39; 95% CI: 1.04–1.86). Polypharmacy and multimorbidity were independently associated with the composite outcome. **Conclusions**: Medication safety gaps were common but differed by care setting. Outpatient care showed more high-risk or insufficiently reviewed prescribing, whereas inpatient care showed more therapeutic omissions. These findings support prospective evaluation of medication review, reconciliation, deprescribing, and reassessment of omitted therapies in similar public referral settings.

## 1. Introduction

Potentially inappropriate medication use has become a major medication safety concern in older adults, particularly in settings where multimorbidity, long-term pharmacotherapy, and fragmented follow-up converge; a worldwide outpatient meta-analysis including 94 studies, 132 prevalence estimates, and approximately 371.2 million older adults reported a pooled prevalence of potentially inappropriate medication use of 36.7%, increasing to 46.9% in South America [1]. The 2023 American Geriatrics Society (AGS) Beers Criteria provide the most recent international update for identifying potentially inappropriate medications in adults aged 65 years or older [2]. In the present study, however, medication classification was based on the Argentine-adapted Spanish-language version of the 2019 AGS Beers Criteria because it was the most recent formally published regional adaptation available when the data-extraction protocol was established [3,4]. The Screening Tool of Older Persons’ Prescriptions (STOPP) and the Screening Tool to Alert to Right Treatment (START), version 3, complement this approach by distinguishing potentially inappropriate prescribing from potential prescribing omissions and include 133 STOPP and 57 START criteria validated through an international Delphi consensus [5]. In hospitalized older adults, potentially inappropriate prescribing has been associated with higher odds of adverse-drug-event-related admissions adjusted odds ratio (AOR) of 1.91, functional decline (AOR 1.60), and adverse drug reactions or events (AOR 1.26) [6]. Together, these findings position inappropriate pharmacotherapy as both a prescribing-quality problem and a patient-safety outcome.

Reported prevalence estimates vary considerably according to the screening instrument, study population, and care setting [1,7]. Studies applying the Beers or STOPP/START criteria have documented substantial burdens of potentially inappropriate prescribing in both outpatient and hospital populations, but direct comparisons of potentially inappropriate medications and prescribing omissions across both settings within the same health system remain scarce [8,9]. This gap supports a setting-specific assessment of high-risk prescribing and therapeutic omission.

Care setting may influence prescribing quality through differences in clinical priorities and medication-management processes. In outpatient care, treatment is generally oriented toward long-term disease control, and medications may be continued across repeated visits without systematic reassessment of treatment duration, therapeutic duplication, cumulative sedative or anticholinergic burden, or changes in the risk–benefit balance. Hospitalization, in contrast, may provide opportunities for medication reconciliation, closer clinical monitoring, and discontinuation of unnecessary or high-risk treatments. However, acute stabilization may take precedence over chronic preventive care, and some indicated medications may be temporarily withheld, postponed, or not restarted before discharge. Transitions between outpatient and inpatient care may further introduce medication discrepancies when histories are incomplete or when treatment changes are not communicated effectively. These mechanisms provide a clinical basis for expecting different profiles of potentially inappropriate prescribing and prescribing omissions across care settings [6,8,10].

Juliaca provides an informative setting for evaluating medication safety in the Peruvian Andes. Hospital Carlos Monge Medrano is a public category II-2 general referral hospital located at approximately 3877 m above sea level and serving more than 300,000 people from San Román and neighboring provinces. Its referral role brings together older adults from urban, peri-urban, and rural areas whose continuity of care and access to medicines may be shaped by geographic barriers, interfacility referrals, and medication availability within the public health system. These characteristics may produce prescribing patterns that differ from those reported in coastal metropolitan hospitals or highly specialized geriatric services [11].

A targeted search of PubMed and SciELO through July 2026 identified peer-reviewed Peruvian articles addressing inappropriate medication use and medication-related harm in older adults [12,13,14], but no published article that simultaneously evaluated Beers-defined potentially inappropriate medications, STOPP-defined potentially inappropriate prescribing, and START-defined potential prescribing omissions across outpatient and inpatient care. This study aimed to compare medication safety outcomes between older adults receiving outpatient and inpatient care in Juliaca, Peru, using the Beers and STOPP/START criteria. Based on the differences in clinical priorities and medication-management processes between care settings, we hypothesized that outpatient care would show a higher prevalence of Beers-defined potentially inappropriate medications and STOPP-defined potentially inappropriate prescribing, whereas hospitalization would show a higher prevalence of START-defined potential prescribing omissions. We further hypothesized that polypharmacy and multimorbidity would be associated with a higher prevalence of overall medication safety gaps. For sample-size planning, the study was powered using the expected prevalence of potentially inappropriate prescribing, with a higher prevalence anticipated in outpatient than in inpatient care.

## 2. Materials and Methods

### 2.1. Study Design and Setting

We conducted an observational, analytical, cross-sectional study based on a retrospective review of medical records of older adults in Peru. The study was conducted at Hospital Carlos Monge Medrano, a public category II-2 general referral hospital located in Juliaca, Puno, at 3877 m above sea level. The hospital has 165 beds and provides outpatient, inpatient, emergency, intensive care, surgical, diagnostic, rehabilitation, dialysis, and pharmacy services to the population served by the San Román Health Network [11]. As part of the public health system, it primarily serves patients receiving care through the regional public-sector network, including beneficiaries of the Seguro Integral de Salud; however, individual insurance status and socioeconomic information were not available in the study database. The outpatient stratum comprised scheduled ambulatory visits conducted in hospital-based general and specialty clinics, including internal medicine, cardiology, neurology, pulmonology, gastroenterology, nephrology, urology, surgery, and traumatology. These encounters did not involve hospital admission and were distinct from community-level primary-care services. For each outpatient participant, medication exposure was assessed using the active prescription documented at the selected hospital clinic visit. The study period included records of older adults who received outpatient care or were hospitalized between January and December 2024.

The study was designed to characterize medication safety gaps across outpatient and inpatient care, including both high-risk prescribing and clinically relevant prescribing omissions. Inappropriate pharmacotherapy was assessed using two explicit, criteria-based screening tools: the Argentine-adapted version of the 2019 American Geriatrics Society (AGS) Beers Criteria [3,4] and the 2023 STOPP/START criteria (version 3) [5]. The analytical approach considered care setting as the main exposure. Beers-defined potentially inappropriate medication, STOPP-defined potentially inappropriate prescribing, and START-defined potential prescribing omission were evaluated as separate primary outcomes, while overall medication safety gaps were examined as a secondary exploratory composite outcome.

### 2.2. Study Population, Sample Size, and Sampling

The study population consisted of medical records of adults aged 60 years or older who received at least one prescribed medication and were treated either in outpatient care or during hospitalization at Hospital Carlos Monge Medrano during 2024. In Peru, Law No. 30490 and the national technical standard for the comprehensive care of older persons define an older adult as a person aged 60 years or older; accordingly, this threshold was used to define the study population [15,16]. The target population was stratified according to care setting, with 851 potentially eligible inpatient records and 743 potentially eligible outpatient records.

The sample size was calculated using OpenEpi version 3 and the Fleiss formula with continuity correction for the comparison of two independent proportions. The calculation compared an expected prevalence of 84.6% in outpatient care with 63.8% in inpatient care, corresponding to a target absolute difference of 20.8 percentage points. The outpatient estimate was obtained from a 2023 Peruvian undergraduate thesis that reviewed 310 medical records of older adults treated in outpatient, inpatient, and emergency services at a level II public hospital in Huaraz and reported an 84.6% prevalence of STOPP-defined potentially inappropriate prescribing among outpatients [17]. The inpatient estimate was obtained from a 2025 Peruvian undergraduate thesis involving 127 hospitalized older adults at Hospital Apoyo Iquitos and reported a 63.8% prevalence of potentially inappropriate prescribing, defined as the presence of at least one positive STOPP/START criterion [18].

Assuming a two-sided 95% confidence level, 80% statistical power, and an outpatient-to-inpatient allocation ratio of 0.87, the minimum required sample size was 157 medical records. Because these estimates originated from separate populations and differed in their operational definitions, they were used as pragmatic assumptions for sample-size planning in the absence of directly comparable Peruvian cross-setting estimates, rather than as directly comparable epidemiological estimates.

The sampling frame consisted of separate nominal lists of older adults recorded in the hospital’s outpatient and inpatient registers between January and December 2024. These lists were obtained through the hospital’s Statistics and Informatics Unit. No additional restrictions based on medical specialty, day of attendance or admission, weekend care, or length of stay were applied beyond the prespecified eligibility criteria. Within each stratum, a random number was assigned to every record using Microsoft Excel for Microsoft 365 (Version 2509), and the records were sorted in ascending order according to the generated number.

Records were reviewed following this random order until the required sample size for each stratum was reached. A total of 194 medical records were assessed, of which 37 (19.1%) were excluded because they were incomplete, illegible, unavailable for review, or lacked sufficient clinical or medication information to apply the Beers and STOPP/START criteria. Review then continued with the next record in the randomly ordered list. The final analytical sample comprised 157 records, including 73 from outpatient care and 84 from hospitalization. Because the excluded records lacked sufficiently complete and reliable demographic and clinical information, a formal comparison between included and excluded records was not feasible.

### 2.3. Eligibility Criteria

Medical records were eligible if they corresponded to patients aged 60 years or older, of either sex, who had been treated at Hospital Carlos Monge Medrano between January and December 2024, either in outpatient care or hospitalization. Records were required to be complete and legible, and to contain sufficient information on clinical diagnoses and prescribed medications to allow application of the Beers and STOPP/START criteria.

Records were excluded if they corresponded to patients with active cancer, patients receiving chemotherapy or radiotherapy during the study period, patients in terminal condition, or patients receiving palliative care. Records were also excluded if the patient died during hospitalization, was referred or transferred to another health facility for continuation of care, had voluntary discharge that prevented adequate assessment of the pharmacotherapeutic regimen, or had incomplete or illegible clinical information.

### 2.4. Variables and Definitions

The main exposure variable was care setting, defined as the type of clinical setting in which the prescription was generated or recorded. Care setting was categorized as outpatient care or hospitalization.

The primary outcome variables were Beers-defined potentially inappropriate medications, STOPP-defined potentially inappropriate prescribing, and START-defined potential prescribing omissions. Overall medication safety gaps were evaluated as a secondary exploratory composite outcome. Beers-defined potentially inappropriate medication was defined as the presence of at least one prescribed medication classified as potentially inappropriate according to the Argentine-adapted version of the 2019 AGS Beers Criteria [3,4]. STOPP-defined potentially inappropriate prescribing was defined as the presence of at least one medication-related problem identified using the STOPP section of the 2023 STOPP/START criteria. START-defined potential prescribing omission was defined as the absence of at least one clinically indicated medication or therapeutic class according to the START section of the 2023 STOPP/START criteria.

Overall medication safety gaps were defined as the presence of at least one Beers-defined potentially inappropriate medication, STOPP-defined potentially inappropriate prescribing event, or START-defined potential prescribing omission. This secondary exploratory composite was used as a broad summary indicator of whether any explicit medication safety concern was identified in a medical record, regardless of whether it reflected high-risk medication exposure, inappropriate continuation or duplication, or omission of an indicated therapy. The composite was not intended to imply that the three components represent the same clinical process, have equivalent severity, or produce comparable clinical consequences. Accordingly, Beers-, STOPP-, and START-defined outcomes were also analyzed and interpreted separately, while the composite was used only to summarize the overall burden of having at least one identified prescribing-quality concern.

Covariates included sex, age group, hypertension, type 2 diabetes mellitus, chronic kidney disease, cardiovascular disease, cognitive impairment or dementia, history of falls during the previous year, multimorbidity, number of prescribed medications, polypharmacy, and hyperpolypharmacy. Sex and age were extracted from the demographic information in the medical record. Hypertension, type 2 diabetes mellitus, chronic kidney disease, cardiovascular disease, and cognitive impairment or dementia were coded as present only when explicitly documented by a treating clinician in the problem list, medical history, clinical assessment, or discharge diagnosis; these conditions were not inferred from medication use alone. Chronic kidney disease was not assigned solely on the basis of a single serum creatinine or estimated glomerular filtration rate measurement. Cognitive impairment or dementia was based on a documented clinical diagnosis because standardized cognitive scores, such as the Mini-Mental State Examination or Montreal Cognitive Assessment, were not consistently available; no diagnosis was retrospectively inferred from nonspecific symptoms or psychotropic medication use. History of falls was coded as present when at least one fall during the preceding 12 months was explicitly documented in the medical history or clinical notes; no formal fall-risk scale was used. Multimorbidity was defined using a simple, unweighted count of two or more physician-documented chronic conditions [19]; the Charlson Comorbidity Index was not calculated.

For outpatients, the number of prescribed medications was based on the active regimen documented at the selected hospital clinic visit. For hospitalized patients, medication exposure was assessed longitudinally across the entire index hospitalization, from admission to discharge, using all medication orders and pharmacological treatments documented in the medical record. Distinct active ingredients or fixed-dose combinations prescribed at any point during the hospital stay were included, while duplicate entries of the same active ingredient were counted once. Scheduled and as-needed medications were included, as were oral, inhaled, intravenous, and subcutaneous formulations. Duplicate entries of the same active ingredient were counted once. No topical preparations were recorded in the eligible medication lists and, therefore, none contributed to the medication count. Polypharmacy and hyperpolypharmacy were defined as five or more and ten or more prescribed medications, respectively [20]. Medication exposure was determined from the active prescriptions documented in the medical record at the index outpatient encounter or during the index hospitalization. Dispensing records, medication refill data, pill counts, and validated adherence measures were not consistently available; therefore, the study assessed prescribed pharmacotherapy rather than confirmed medication use or adherence.

### 2.5. Data Collection Procedures

Data were collected through structured documentary review of medical records. A standardized data extraction form was used to record sociodemographic characteristics, clinical diagnoses, history of falls, care setting, number of prescribed medications, and all pharmacological treatments documented in the medical record.

The medical records were also reviewed to determine, when documented, the original prescribing setting and timing of the identified medication safety problems. However, the records did not consistently indicate whether potentially inappropriate medications used in outpatient care had been initiated in primary care, a hospital specialty clinic, or a previous hospitalization. Similarly, inpatient records did not reliably distinguish between potential prescribing omissions that were already present before admission and those that arose during hospitalization. Because these data could not be classified consistently across participants, the source and timing of potentially inappropriate medications (PIMs) and potential prescribing omissions (PPOs) were not included as formal study variables.

For hospitalized patients, the pharmacotherapeutic assessment covered the full trajectory of the index hospital stay, from admission to discharge. All documented medication orders, treatment modifications, discontinuations, temporary withholding decisions, and relevant clinical information recorded during the hospitalization were reviewed. Beers and STOPP/START criteria were applied according to the medication regimen and clinical information available at the time each criterion became applicable, rather than at a single fixed time point. For outpatients, the assessment was based on the active medication regimen documented at the selected clinic visit.

The Beers and STOPP/START criteria were applied retrospectively to the pharmacotherapeutic information available in each medical record. The Beers assessment was based on the Argentine-adapted Spanish-language version of the 2019 AGS Beers Criteria developed by Calabro et al. through a Delphi consensus process [3] and derived from the original 2019 AGS Beers Criteria [4]. This adaptation revised the original list according to medication availability and prescribing practices in Argentina. It was not specifically adapted or validated for Peru, and the research team made no additional changes to the medication lists, clinical thresholds, renal-dosing guidance, or recommendations. STOPP/START was applied using the peer-reviewed Spanish version of version 3 published by Delgado-Silveira et al., which was derived from the original 2023 international consensus criteria [5,21]. Because formally published Spanish-language versions of both tools were used, no study-specific translation, back-translation, or linguistic pilot testing was required. The Spanish criteria were applied without local modification. At the time the data-extraction protocol was established, STOPP/START version 3 was the most recent available version, while the Argentine-adapted 2019 Beers Criteria represented the most recent formally published Spanish-language regional adaptation available to the investigators. Because Peruvian regulations define older adults as persons aged 60 years or older, both tools were applied to the full study population. Nevertheless, as these criteria were originally developed mainly for adults aged 65 years or older, findings involving participants aged 60–64 years should be interpreted cautiously. The standardized data extraction form served only as an operational tool for organizing information obtained from the medical records and did not constitute an independent measurement scale.

The full set of 133 STOPP and 57 START criteria from version 3 was initially reviewed for applicability to each medical record. Each item was classified as applicable, not applicable, or not assessable based on the documented diagnoses, active medication regimen, dose, route, frequency, treatment duration, relevant clinical history, and available laboratory or diagnostic findings. For STOPP, a criterion was recorded as positive only when all medication- and condition-specific requirements were documented. Treatment duration was determined from prescription dates, start and stop dates when available, inpatient medication orders, and repeated prescriptions or follow-up entries; duration-dependent criteria were not scored when duration could not be established reliably. For START, a potential prescribing omission was recorded only when the qualifying diagnosis or clinical condition was documented, the recommended medication or therapeutic class was absent from the active regimen, and no contraindication, previous intolerance, or documented clinical reason for non-prescription was identified. Criterion-specific laboratory information, including serum creatinine or estimated glomerular filtration rate when renal function was required, was used when available. Missing documentation was not interpreted as the absence of a diagnosis, indication, contraindication, or treatment, and non-assessable criteria did not contribute to the outcome numerator. Vaccination-related START criteria were excluded from the quantitative analysis because vaccination status was not consistently available in the hospital records and may have been documented in other healthcare facilities; no other criterion was excluded a priori.

As a sensitivity analysis, we conducted a patient-level, update-specific crosswalk to determine whether changes introduced in the 2023 AGS Beers Criteria would materially alter the original 2019 Beers classification. We examined all criteria added or materially modified between the 2019 and 2023 versions that were relevant to medications or clinical conditions represented in the medication-level database, including aspirin for primary prevention, the expanded sulfonylurea criterion, and the addition of anticholinergic medications to the history-of-falls or fractures criterion. The resulting patient-level classification was then compared with the original classification based on the Argentine-adapted 2019 criteria.

Data extraction and pharmacotherapy classification were performed independently by two trained reviewers using the same standardized data extraction form and predefined operational criteria. Before formal data collection, both reviewers completed a standardization session in which they reviewed the definitions and application rules for the Beers, STOPP, and START criteria to ensure a consistent approach. Each medical record was independently evaluated for Beers-defined potentially inappropriate medications, STOPP-defined potentially inappropriate prescribing, START-defined potential prescribing omissions, and the composite medication safety outcome. The reviewers were aware of the study objective and expected direction of the comparison; however, all classifications were completed independently before the comparative statistical analyses were conducted. Blinding to care setting was not feasible because outpatient and inpatient records could be distinguished from their structure and clinical content. No disagreements occurred between the reviewers, corresponding to 100% agreement and Cohen’s kappa values of 1.00 for all classifications. Therefore, no adjudication was required. After completion of the independent review, the extraction forms were checked for consistency, and the database was coded, cleaned, and examined for missing or inconsistent values before statistical analysis.

### 2.6. Statistical Analysis

Categorical variables were summarized using absolute and relative frequencies. The number of prescribed medications was summarized using the median and interquartile range because of its non-normal distribution. Comparisons between outpatient and hospitalized patients were performed using Pearson’s chi-square test for categorical variables and the Mann–Whitney U test for non-normally distributed numerical variables. The numbers of Beers-, STOPP-, and START-defined medication-related problems identified per participant were summarized using the median, interquartile range, and observed range because these count variables were right-skewed and included zero values. Between-setting comparisons were performed using the Mann–Whitney U test.

The prevalence of each primary outcome was estimated for the overall sample and according to care setting; the secondary composite outcome was analyzed separately as an exploratory summary measure. Pearson’s chi-square test was used to compare the prevalence of Beers-defined potentially inappropriate medication, STOPP-defined potentially inappropriate prescribing, START-defined potential prescribing omission, and overall medication safety gaps between outpatient and hospitalized patients.

Crude and adjusted prevalence ratios (PRs) with 95% confidence intervals (CIs) were estimated using modified Poisson regression with a robust sandwich variance estimator [22]. This approach was selected because the outcomes were common in the study population and prevalence ratios provide a more directly interpretable measure of association than odds ratios in cross-sectional studies. The robust variance estimator was used to account for variance misspecification resulting from fitting a Poisson model to binary outcomes. Modified Poisson regression was preferred over log-binomial regression because it provides comparable prevalence-ratio estimates while being less susceptible to convergence problems.

For the association between care setting and each medication safety outcome, outpatient care was used as the reference category. The adjustment set age group, sex, polypharmacy, and multimorbidity was specified a priori based on clinical relevance, previous evidence, and their potential roles as confounders of the association between care setting and medication safety outcomes. Variables were not selected using automated stepwise procedures or solely according to bivariable *p*-values. Polypharmacy and multimorbidity were included simultaneously because they represent related but clinically distinct dimensions of prescribing complexity: medication burden and chronic disease burden, respectively. Their joint inclusion was retained only after confirming that the collinearity diagnostics did not indicate relevant model instability. These outcome-specific models were designed to estimate the adjusted association between care setting and each primary medication safety outcome. The remaining covariates were included for confounding control and were not treated as candidate predictors or interpreted as independent risk factors because the study was not designed or powered for multivariable risk-factor profiling for each criterion-specific outcome.

An additional multivariable modified Poisson regression model with robust variance was fitted to identify factors associated with overall medication safety gaps. This model included care setting, sex, age group, polypharmacy, multimorbidity, and chronic kidney disease. Chronic kidney disease was included because of its direct clinical relevance to medication selection, dosing, and prescribing safety in older adults. Multicollinearity was assessed using variance inflation factors (VIFs), with values greater than 5 considered indicative of potentially relevant collinearity. All statistical tests were two-sided, and statistical significance was set at *p* < 0.05. Because several related medication safety outcomes and comparisons were evaluated, no formal adjustment for multiple testing was applied. Given the absence of formal multiplicity adjustment, findings were interpreted cautiously and primarily according to the magnitude and precision of the prevalence ratios and their 95% confidence intervals rather than solely according to whether *p*-values crossed the 0.05 threshold.

Exploratory interaction terms between care setting and age ≥80 years, multimorbidity, or polypharmacy were considered but were not fitted because the available subgroup sizes, particularly the small number of outpatients aged ≥80 years, would have produced unstable estimates and insufficient statistical precision. Additional interaction testing would also have increased model complexity and the risk of type I error in this relatively small sample.

Sensitivity analyses were conducted to assess the robustness of the adjusted estimates. First, missingness was evaluated for all outcomes and covariates included in the regression models. Because all 157 records in the analytical sample had complete information for these variables, the complete-case analysis included the full sample and no data imputation was required. Second, all adjusted models were re-estimated using hyperpolypharmacy, defined as ten or more prescribed medications, instead of polypharmacy, defined as five or more medications. Polypharmacy and hyperpolypharmacy were not entered simultaneously because they represent nested measures of medication burden. All other adjustment variables, model specifications, and reference categories remained unchanged.

Model adequacy was examined using the Pearson chi-square and deviance statistics divided by their corresponding residual degrees of freedom. Values greater than 1 were considered suggestive of overdispersion. Because the outcomes were binary and therefore did not satisfy the Poisson mean–variance assumption exactly, the robust sandwich variance estimator was retained regardless of the dispersion statistics to provide standard errors that were less sensitive to variance misspecification. Each participant contributed only one medical record to the analysis. Prescriber- and ward-level identifiers were not available in the study database; therefore, cluster-robust standard errors and multilevel models based on these units could not be estimated. Medical specialty was not used as a clustering variable because it did not uniquely identify either the prescribing clinician or the hospital unit.

All analyses were performed using IBM SPSS Statistics version 26. Pearson’s chi-square tests were conducted using the CROSSTABS procedure, and Mann–Whitney U tests were performed using the NPTESTS procedure. Crude and adjusted prevalence ratios were estimated using the GENLIN procedure, specifying a Poisson probability distribution, log link function, and robust covariance estimator. Exponentiated regression coefficients were reported as prevalence ratios with 95% confidence intervals. Variance inflation factors and tolerance statistics were obtained using the linear REGRESSION procedure with collinearity diagnostics applied to the same sets of adjustment variables included in the modified Poisson models. No user-written macros or external SPSS extensions were required. The SPSS syntax used for data management and statistical analysis is available from the corresponding author upon reasonable request.

### 2.7. Ethical Considerations

The study was conducted in accordance with the ethical principles of the Declaration of Helsinki and applicable Peruvian regulations for health research. The protocol was reviewed and approved by the Institutional Committee of Ethics and Scientific Integrity of Universidad Privada Norbert Wiener, Lima, Peru, under approval file No. 3863-2025, issued on 31 December 2025. This approval specifically covered the retrospective review of medical records corresponding to care provided between January and December 2024. Although the records preceded the ethics approval, no medical records were accessed and no study data were extracted before ethical authorization was obtained.

Following ethics approval, formal institutional authorization to conduct the study and access the required medical records was granted by the Teaching and Research Support Unit of the San Román Health Network and Hospital Carlos Monge Medrano on 26 January 2026. Data collection began only after both the university ethics approval and the hospital authorization had been obtained. The hospital authorization covered access to existing clinical records for the approved research purpose; no intervention, patient contact, or modification of clinical care occurred.

The study involved minimal risk and used only previously recorded clinical information. Individual informed consent was not required because no participants were recruited, interviewed, or exposed to any study-related procedure. Each medical record was assigned a coded study identifier, and names, identity-document numbers, addresses, and other direct identifiers were not included in the analytical database. Access to the coded database was restricted to the research team, the data were used exclusively for the approved study, and all findings were reported in aggregate form to prevent individual identification.

## 3. Results

Of the 194 medical records assessed, 37 (19.1%) were excluded because of incomplete or illegible documentation, leaving 157 records in the final analytical sample: 73 from outpatient care and 84 from hospitalization. The baseline characteristics of the study population are summarized in Table 1. The two care-setting groups were broadly comparable in their sociodemographic and clinical characteristics. Hospitalized patients had a higher median number of prescribed medications than outpatients [5 (IQR: 4–8) vs. 4 (IQR: 3–6), *p* = 0.012], while the difference in polypharmacy prevalence did not reach the conventional threshold for statistical significance (52.4% vs. 37.0%, *p* = 0.053).

Inter-rater agreement was complete for all pharmacotherapy classifications. The two reviewers reached 100% agreement for Beers-defined potentially inappropriate medications, STOPP-defined potentially inappropriate prescribing, START-defined potential prescribing omissions, and overall medication safety gaps, with Cohen’s κ = 1.00 for each outcome.

Medication safety outcomes according to care setting are presented in Table 2. Overall, 124 of 157 participants (79.0%) had at least one identified medication safety gap. Compared with outpatient care, hospitalization showed a lower prevalence of STOPP-defined potentially inappropriate prescribing and a higher prevalence of START-defined potential prescribing omissions. Beers-defined potentially inappropriate medication was more frequent in outpatient care, although the between-setting estimate was imprecise, whereas the prevalence of the composite outcome was similar in both settings.

Beyond the binary prevalence outcomes, the number of criterion-specific medication-related problems identified per participant also differed by care setting (Table 3). Outpatients had higher numbers of Beers-defined potentially inappropriate medications [median: 1 (IQR: 0–2) vs. 0 (IQR: 0–1), *p* = 0.014] and STOPP-defined potentially inappropriate prescribing problems [median: 1 (IQR: 0–2) vs. 0 (IQR: 0–1), *p* = 0.009] than hospitalized patients. In contrast, hospitalized patients had a higher number of START-defined potential prescribing omissions [median: 1 (IQR: 0–2) vs. 0 (IQR: 0–1), *p* = 0.006]. These count-based findings were consistent with the direction of the corresponding prevalence comparisons.

In the update-specific sensitivity analysis, the changes introduced in the 2023 AGS Beers Criteria did not alter patient-level classification. The same 73 of 157 participants (46.5%) were classified as having at least one Beers-defined potentially inappropriate medication under both versions, with no newly positive or no longer positive cases and complete patient-level agreement (Cohen’s κ = 1.00). One participant met the newly added criterion involving a history of falls and anticholinergic medication use but had already been classified as Beers-positive under the 2019 criteria. All aspirin prescriptions were documented for secondary cardiovascular prevention, and glibenclamide was the only sulfonylurea identified; therefore, the updated aspirin and sulfonylurea recommendations resulted in no additional reclassification.

The adjusted associations between care setting and medication safety outcomes are summarized in Table 4. Hospitalization was associated with a lower prevalence of STOPP-defined potentially inappropriate prescribing (adjusted PR = 0.74; 95% CI: 0.55–0.99) and a higher prevalence of START-defined potential prescribing omissions (adjusted PR = 1.39; 95% CI: 1.04–1.86). The estimate for Beers-defined potentially inappropriate medication also favored a lower prevalence during hospitalization, although its confidence interval included the null value (adjusted PR = 0.76; 95% CI: 0.56–1.02). Care setting was not associated with the composite medication safety outcome (adjusted PR = 1.02; 95% CI: 0.89–1.18). The Beers estimate was interpreted according to its magnitude and precision rather than solely according to the *p*-value.

The three most frequent medication-related problems identified with each screening tool are reported below, while the broader criterion-specific distribution is presented in Supplementary Table S1. According to the Beers Criteria, the most common problems were long-term use of nonsteroidal anti-inflammatory drugs in older adults at gastrointestinal or renal risk, identified in 24 participants (15.3%); benzodiazepines or Z-drugs associated with risks of falls, sedation, or cognitive impairment, identified in 21 participants (13.4%); and proton pump inhibitors without a clear long-term indication, identified in 19 participants (12.1%). According to the STOPP criteria, the most frequent problems were medication use beyond the recommended duration without documented reassessment in 29 participants (18.5%), therapeutic duplication within the same pharmacological class in 17 participants (10.8%), and use of medications that increase fall risk among patients with a previous fall or frailty markers in 15 participants (9.6%). According to the START criteria, the leading omissions were statin therapy when indicated in patients with diabetes or established cardiovascular disease in 24 participants (15.3%), antiplatelet therapy when indicated in patients with documented atherosclerotic cardiovascular disease in 19 participants (12.1%), and renin–angiotensin system blockade when indicated in patients with diabetes, hypertension, heart failure, or proteinuric kidney disease in 16 participants (10.2%) (Supplementary Table S1).

In the adjusted analysis, polypharmacy and multimorbidity remained independently associated with overall medication safety gaps. Participants with polypharmacy had a 19% higher adjusted prevalence of the outcome (adjusted PR = 1.19; 95% CI: 1.04–1.36), while those with multimorbidity had a 17% higher adjusted prevalence (adjusted PR = 1.17; 95% CI: 1.01–1.35). The associations observed for age ≥80 years and chronic kidney disease in the crude analyses were attenuated after adjustment, while care setting and sex were not associated with the composite outcome (Table 5).

No relevant multicollinearity was identified in the adjusted models; VIFs ranged from 1.05 to 1.31, including in models containing both polypharmacy and multimorbidity. These values supported retaining both variables as separate indicators of medication burden and chronic disease complexity. Across the adjusted models, the Pearson chi-square-to-degrees-of-freedom ratios ranged from 0.22 to 0.57, and the corresponding deviance-to-degrees-of-freedom ratios ranged from 0.36 to 0.70. Because all ratios were below 1, there was no evidence of overdispersion. Robust variance estimates were nevertheless retained because the outcomes were binary and the conventional Poisson variance assumption was not expected to hold exactly.

All outcomes and covariates included in the regression models had complete information for the 157 participants. Consequently, the complete-case sensitivity analysis included the full analytical sample, produced the same estimates as the primary analysis, and did not require data imputation.

When hyperpolypharmacy replaced polypharmacy in the adjusted models, the direction of the associations between care setting and all medication safety outcomes remained consistent. The association with Beers-defined potentially inappropriate medication became statistically significant, whereas the remaining care-setting associations retained the same statistical interpretation as in the primary analysis. Hospitalization was associated with a lower prevalence of Beers-defined potentially inappropriate medication (adjusted PR = 0.69; 95% CI: 0.49–0.98; *p* = 0.036) and STOPP-defined potentially inappropriate prescribing (adjusted PR = 0.64; 95% CI: 0.46–0.88; *p* = 0.007), as well as a higher prevalence of START-defined potential prescribing omissions (adjusted PR = 1.40; 95% CI: 1.01–1.94; *p* = 0.046). Care setting remained unassociated with overall medication safety gaps (adjusted PR = 0.96; 95% CI: 0.82–1.13; *p* = 0.628).

Hyperpolypharmacy was associated with STOPP-defined potentially inappropriate prescribing (adjusted PR = 1.62; 95% CI: 1.07–2.44; *p* = 0.022), but not with Beers-defined potentially inappropriate medication, START-defined potential prescribing omissions, or overall medication safety gaps. This suggests that the association observed with the broader polypharmacy definition was not reproduced when medication burden was restricted to the higher threshold of ten or more medications. Multimorbidity remained associated with overall medication safety gaps in the sensitivity model (adjusted PR = 1.32; 95% CI: 1.10–1.59; *p* = 0.003). The key sensitivity estimates are presented in Supplementary Table S2.

## 4. Discussion

In this cross-sectional study of older adults receiving outpatient or inpatient care in a Peruvian referral hospital, medication safety gaps were highly prevalent and affected nearly four out of five participants. The main finding was not only the high frequency of inappropriate pharmacotherapy, but also the persistence of different medication safety gaps across the continuum of care. Outpatient care showed a greater burden of Beers-defined potentially inappropriate medications and STOPP-defined potentially inappropriate prescribing, suggesting chronic exposure to high-risk, prolonged, duplicated, or insufficiently reviewed medications. In contrast, hospitalization was independently associated with a higher prevalence of START-defined potential prescribing omissions, indicating that inpatient care may reduce some forms of high-risk prescribing while leaving important indicated therapies unaddressed. Overall medication safety gaps did not differ meaningfully between outpatient and inpatient care, suggesting that medication-related risk is not confined to one setting but changes in profile according to the point of care. Polypharmacy and multimorbidity remained independently associated with overall medication safety gaps, reinforcing that medication safety in older adults is driven by treatment complexity and chronic disease burden rather than by care setting alone.

The composite outcome should be interpreted as a summary of the presence of any identified prescribing-quality concern rather than as a single homogeneous clinical endpoint. Beers-defined potentially inappropriate medications, STOPP-defined potentially inappropriate prescribing, and START-defined potential prescribing omissions reflect distinct clinical processes and may differ in mechanism, severity, preventability, and potential consequences. Combining them increased sensitivity for describing the overall burden of medication safety concerns, but it may also obscure opposing component-specific patterns. For example, the similar prevalence of the composite outcome across care settings coexisted with lower STOPP-defined inappropriate prescribing and higher START-defined omissions during hospitalization. Therefore, the component-specific findings provide the most clinically informative comparison between outpatient and inpatient care.

The most clinically informative finding was the divergence between care settings rather than the overall prevalence alone. Although the composite outcome was similarly frequent in outpatient and inpatient care, hospitalization was associated with fewer STOPP-defined prescribing problems but more START-defined potential prescribing omissions. This pattern suggests that hospital admission may provide an opportunity to discontinue, modify, or reassess some prolonged, duplicated, or high-risk treatments without necessarily ensuring that all indicated chronic therapies are initiated or resumed. The coexistence of inappropriate prescribing and therapeutic omission has also been reported in hospitalized older populations [23,24], but the present study adds a direct comparison between outpatient and inpatient records within the same institution.

The higher outpatient burden of Beers- and STOPP-defined problems may reflect the persistence of long-term prescriptions across repeated visits, particularly when treatment duration, therapeutic duplication, fall risk, anticholinergic burden, or continued indication are not systematically reassessed. This interpretation is consistent with evidence showing that inappropriate medication use in outpatient and community settings is closely related to medication burden and the screening criteria applied [1,9,25]. However, the available records did not establish whether the identified medications had originally been initiated in primary care, a hospital specialty clinic, or a previous hospitalization. The outpatient findings should therefore be interpreted as accumulated prescribing problems observed at that point of care, rather than as proof that the outpatient service originated every identified PIM or PIP.

The original AGS Beers Criteria were developed primarily for the United States, where available medicines, therapeutic alternatives, formularies, and prescribing patterns may differ from those of the Peruvian public health system. We therefore used the Argentine-adapted Spanish-language version of the 2019 criteria because it was more regionally and linguistically relevant, although it has not been specifically validated for Peru. The absence of patient-level reclassification in the sensitivity analysis using the 2023 AGS update supports the stability of the prevalence estimate in this sample, but it does not establish full cultural or clinical equivalence of the criteria. The higher outpatient prevalence observed in this study may indicate that long-term prescriptions persist without structured geriatric review. Within this setting, the finding supports strengthening medication review in ambulatory care, where prolonged exposure to high-risk pharmacotherapy may accumulate over time.

The higher prevalence of START-defined potential prescribing omissions during hospitalization may reflect both genuine therapeutic gaps and temporary clinical decisions made during acute care. Hospital teams often prioritize stabilization and may postpone, withhold, or reassess chronic preventive therapies because of planned procedures, acute instability, renal-function changes, or temporary contraindications. Similar burdens of potential prescribing omissions have been reported in hospitalized older populations [23,24,26]. However, because the medical records did not reliably distinguish pre-existing omissions from temporary inpatient decisions, START-positive findings in this study should be interpreted as prompts for medication reconciliation and therapeutic reassessment before discharge, rather than as direct evidence of poor inpatient prescribing.

Polypharmacy was not only frequent in this study but also functioned as an independent marker of inappropriate pharmacotherapy. The overall prevalence of 45.2% was similar to the 48% pooled prevalence reported by Tian et al. [1] in older Chinese patients, although their analysis showed a much sharper setting gradient, with 73% among inpatients and 23% among outpatients. The association observed in our study was directionally consistent with their finding that polypharmacy increased the risk of PIM use by more than twofold (RR = 2.03). Similarly, Doherty et al. [25] showed a strong dose–response relationship, with adjusted odds ratios of 3.70 for 5–9 medications and 9.03 for ≥10 medications in relation to STOPP PIMs. Zhu et al. [26] also identified the number of medications as the strongest predictor of PIM use across Beers, STOPP, START, and Chinese criteria. The smaller magnitude in our adjusted model may reflect the use of prevalence ratios, a mixed outpatient–inpatient population, and adjustment for multimorbidity. Clinically, polypharmacy should not be interpreted merely as medication count, but as a warning signal for structured pharmacotherapeutic review.

Multimorbidity was another independent factor associated with inappropriate pharmacotherapy, supporting the idea that clinical complexity drives prescribing vulnerability in older adults. In our study, multimorbidity was present in 59.2% of participants and remained associated with inappropriate pharmacotherapy after adjustment. This finding is consistent with the MoPIM cohort, where older hospitalized patients had a median of eight chronic conditions and showed very high prevalences of PIP, PIM, and PPO [23]. It also aligns with Doherty et al. [25], who reported that START PPOs were particularly associated with increasing age and the presence of three or more chronic conditions. These findings suggest that multimorbidity increases pharmacotherapeutic risk through several pathways: more medications, more prescribers, disease-specific guidelines that compete with one another, and higher probability of both unnecessary continuation and clinically relevant omission. The association observed in our study therefore reflects not only pharmacological exposure, but also fragmentation in care planning for older adults with complex chronic disease. Within this setting, the findings suggest that medication review may be more informative when it considers multimorbidity and competing treatment priorities rather than individual diseases in isolation.

Age ≥ 80 years and chronic kidney disease were clinically relevant but lost statistical significance after adjustment, suggesting that their effect may be partly explained by therapeutic burden and multimorbidity. This differs from Doherty et al. [25], who found that age ≥ 75 years was independently associated with STOPP-defined PIMs, and from Tian et al. [1], who reported a 41.9% pooled prevalence of PIM use among outpatients aged ≥ 80 years. It also contrasts with Karki et al. [27], who found that chronic kidney disease and polypharmacy significantly contributed to PIM prevalence among elderly inpatients assessed with the 2023 AGS Beers Criteria. The attenuation observed in our adjusted model does not mean that advanced age or chronic kidney disease are clinically irrelevant; rather, it suggests that their statistical effect may operate through greater comorbidity, altered pharmacokinetics, and higher medication exposure. This distinction is important because interventions based only on age or renal disease may miss the broader prescribing context. Within this setting, medication risk assessment may benefit from jointly considering age, renal function, multimorbidity, and polypharmacy rather than relying on any single characteristic.

The findings should be interpreted within the geographic and health-system context of Juliaca. Hospital Carlos Monge Medrano receives older adults from urban, peri-urban, and rural areas of the Peruvian highlands, where long travel distances, interfacility referrals, fragmented follow-up, and variable access to medicines may complicate medication continuity and periodic treatment reassessment. Although altitude itself cannot be assumed to directly explain prescribing quality, the high-altitude Andean setting is closely linked to geographic isolation and unequal access to specialized care. In addition, the prescribing environment of a public referral hospital may be shaped by formulary restrictions, uneven medicine availability, fragmented communication between services, and limited opportunities for structured medication review. These conditions could contribute both to the persistence of familiar high-risk treatments in outpatient care and to the non-initiation or delayed reintroduction of indicated therapies during hospitalization. Because medication-review practices, communication processes, and access to specialist pharmacotherapy support were not measured directly, these mechanisms should be interpreted as plausible contextual explanations rather than confirmed causal pathways [10,11,28,29].

The contrasting patterns across care settings suggest that medication-safety interventions may need to be tailored to the point of care. In hospital-based outpatient clinics, periodic medication review could focus on treatment duration, therapeutic duplication, fall risk, anticholinergic burden, and continued indication, particularly among patients with polypharmacy or multimorbidity. Pharmacist-supported review and structured use of explicit screening criteria may help identify medications requiring reassessment without assuming that every flagged prescription should automatically be discontinued [5,28,29].

During hospitalization, structured medication reconciliation at admission and discharge could be used to document which chronic treatments were continued, discontinued, temporarily withheld, or scheduled for reassessment after discharge. Incorporating a brief STOPP/START-based review into discharge workflows, together with pharmacist, geriatric, or clinical-pharmacology consultation for patients with complex regimens, may help address both high-risk prescribing and omitted indicated therapies. Targeted continuing education and locally adapted prescribing protocols may also support more consistent medication review. Because the present study did not evaluate the effectiveness, feasibility, or cost of these strategies, they should be considered candidate interventions for prospective evaluation rather than direct practice recommendations [10,28,29].

This study has several strengths that partly mitigate the limitations inherent to a retrospective single-center design. First, the use of explicit and internationally recognized Beers, STOPP, and START criteria provided standardized definitions for identifying both high-risk prescribing and potential prescribing omissions, thereby reducing classification heterogeneity despite the dependence on medical-record documentation. Second, pharmacotherapy classification was performed independently by two trained reviewers using predefined operational rules, with complete inter-rater agreement. This procedure reduced reviewer-related variability, although it could not eliminate misclassification caused by information that had not been documented in the records. Third, stratified random sampling within outpatient and inpatient care reduced the likelihood of systematic record selection within the available hospital sampling frame and enabled direct comparison across two clinically distinct points of care. Finally, adjusted prevalence ratios and sensitivity analyses using alternative medication-burden definitions allowed the consistency and precision of the main associations to be examined rather than relying solely on unadjusted comparisons or *p*-value thresholds.

However, several limitations should be acknowledged. First, the cross-sectional and retrospective design precluded causal inference and exposed the study to potential information bias because diagnoses, medication indications, treatment duration, contraindications, and laboratory findings depended on the completeness and accuracy of the medical records. This limitation may have particularly affected the application of the STOPP/START criteria, especially the identification of potential prescribing omissions, because an undocumented indication, contraindication, or clinical decision could result in misclassification. To reduce this risk, criteria were recorded as positive only when the required clinical information was documented, non-assessable criteria were not counted as positive, and two reviewers independently applied the same predefined operational rules. These procedures reduced classification heterogeneity, although they could not completely eliminate the effects of incomplete clinical documentation.

Incomplete documentation also prevented reliable attribution of outpatient PIMs to the setting in which they were originally prescribed and prevented distinction between chronic PPOs present before hospital admission and new omissions arising during hospitalization. Consequently, the study identifies where these medication safety gaps were observed, but not necessarily where they originated. Because incomplete documentation could either conceal a genuine prescribing problem or make a clinically appropriate decision appear inappropriate, the direction and magnitude of this potential misclassification cannot be determined.

In addition, some START-defined omissions observed during hospitalization may have represented clinically appropriate temporary withholding or postponement of chronic therapy in response to the acute clinical situation. Because the records did not consistently document the intended duration or rationale for these decisions, the study could not distinguish persistent underprescribing from temporary therapeutic deferral. In addition, hospitalized patients were assessed throughout the entire hospital stay, whereas outpatients were assessed at a single clinic visit. This difference in observation time may have increased the opportunity to identify medication changes, temporary withholding, or prescribing omissions among hospitalized patients and should be considered when comparing the two care settings.

In addition, the composite outcome assigned the same binary weight to clinically different phenomena and did not account for the number, severity, or potential consequences of individual PIMs, PIPs, or PPOs. Its prevalence should therefore be interpreted as the proportion of participants with at least one identified medication safety concern, rather than as a measure of the overall clinical severity of inappropriate pharmacotherapy.

Medication adherence could not be evaluated because the medical records documented prescribed regimens but did not consistently include dispensing information, refill history, pill counts, or validated adherence measures. Consequently, some medications classified as potentially inappropriate may not have been taken as prescribed, while some therapies classified as omitted may have been obtained from another healthcare facility or outside the documented regimen. In addition, the cross-sectional record review did not include follow-up after the index encounter or hospitalization. We therefore could not determine whether the identified medication safety concerns were subsequently corrected or whether they were associated with adverse drug events, falls, readmissions, functional decline, or mortality. The findings should thus be interpreted as prescribing-quality signals rather than evidence of demonstrated medication-related clinical harm.

Second, selection bias is possible because the study was conducted in a single public referral hospital and 37 of the 194 assessed records were excluded because they were incomplete, illegible, unavailable, or lacked sufficient clinical or medication information. A formal comparison between included and excluded records was not possible because the excluded records did not contain sufficiently reliable demographic and clinical information. Therefore, the prevalence estimates may not be generalizable to private facilities, primary-care centers, or hospitals in other Peruvian regions. Accordingly, the findings should be regarded as setting-specific and hypothesis-generating rather than representative of Peru, the Andean region, or low- and middle-income countries more broadly. Nevertheless, records were randomly selected within the outpatient and inpatient strata, and no additional restrictions were applied according to specialty, day of attendance or admission, weekend care, or length of stay. These measures reduced the likelihood of systematic selection within the available hospital sampling frame.

Third, residual confounding cannot be ruled out because potentially relevant factors, including frailty, functional dependence, illness severity, admission diagnosis, admitting specialty, length of hospitalization, previous hospitalizations, the number and complexity of documented diagnoses, socioeconomic conditions, continuity of medication access, prescriber training, and prescriber- or ward-level characteristics, were not consistently available or could not be incorporated into the adjusted models. The sample size also limited the complexity of the multivariable models and increased the possibility of overfitting. For this reason, the study did not undertake post hoc predictor-screening analyses for each criterion-specific outcome. Such analyses would have required outcome-specific causal frameworks, a broader set of clinical covariates, and a larger sample to obtain stable and interpretable estimates.

For the same reason, formal interaction analyses according to age ≥80 years, multimorbidity, and polypharmacy were not performed. The limited numbers within some care-setting subgroups would have yielded imprecise and potentially unstable estimates; therefore, the consistency of the care-setting associations across these clinical subgroups could not be established. In addition, evaluating multiple related outcomes and comparisons may have increased the risk of type I error. Because no multiplicity correction was applied, borderline associations should be interpreted as exploratory and considered alongside their effect sizes and 95% confidence intervals. Prescriber- and ward-level identifiers were unavailable, preventing formal assessment of residual correlation among patients treated by the same clinician or hospital unit. These unmeasured factors may have influenced the magnitude of the adjusted associations, although they do not change the descriptive observation that medication safety gaps were frequent in both care settings.

Although the eligibility threshold of 60 years was consistent with the Peruvian legal and health-sector definition of an older adult, the Beers and STOPP/START criteria were primarily developed for adults aged 65 years or older. Therefore, findings among participants aged 60–64 years should be interpreted cautiously. Finally, explicit screening criteria support systematic medication review but do not replace individualized clinical judgment.

## 5. Conclusions

Among older adults treated at this Peruvian public referral hospital, Beers-, STOPP-, and START-defined medication safety concerns were common but differed by care setting. Outpatient records showed a greater burden of potentially inappropriate medication exposure and prescribing, whereas inpatient records showed more potential prescribing omissions. Polypharmacy and multimorbidity were associated with the secondary composite outcome of overall medication safety gaps.

Because this was a cross-sectional, retrospective, single-center study, these findings should be interpreted as setting-specific associations rather than evidence that care setting caused the observed prescribing patterns or that any particular intervention would improve clinical outcomes. The results identify areas for prospective evaluation, including structured medication review, medication reconciliation, deprescribing, and reassessment of potentially omitted therapies. Larger multicenter studies are needed to determine whether these patterns are reproducible and whether such strategies are feasible, effective, and associated with improved outcomes in other Peruvian healthcare settings.

## Figures and Tables

**Table 1:** Baseline characteristics of the study population according to care setting.

Variable	Category	Overall, *n* = 157	Outpatient Care, *n* = 73	Hospitalization, *n* = 84	*p*-Value
**Sociodemographic characteristics**					
Sex	Male	82 (52.2)	34 (46.6)	48 (57.1)	0.186
	Female	75 (47.8)	39 (53.4)	36 (42.9)	
Age group	60–69 years	66 (42.0)	36 (49.3)	30 (35.7)	0.119
	70–79 years	55 (35.0)	25 (34.2)	30 (35.7)	
	≥80 years	36 (22.9)	12 (16.4)	24 (28.6)	
**Clinical characteristics**					
Hypertension	No	65 (41.4)	34 (46.6)	31 (36.9)	0.220
	Yes	92 (58.6)	39 (53.4)	53 (63.1)	
Type 2 diabetes mellitus	No	103 (65.6)	48 (65.8)	55 (65.5)	0.971
	Yes	54 (34.4)	25 (34.2)	29 (34.5)	
Chronic kidney disease	No	129 (82.2)	64 (87.7)	65 (77.4)	0.093
	Yes	28 (17.8)	9 (12.3)	19 (22.6)	
Cardiovascular disease	No	122 (77.7)	60 (82.2)	62 (73.8)	0.208
	Yes	35 (22.3)	13 (17.8)	22 (26.2)	
Cognitive impairment or dementia	No	138 (87.9)	67 (91.8)	71 (84.5)	0.164
	Yes	19 (12.1)	6 (8.2)	13 (15.5)	
History of falls during the previous year	No	134 (85.4)	66 (90.4)	68 (81.0)	0.095
	Yes	23 (14.6)	7 (9.6)	16 (19.0)	
Multimorbidity	No	64 (40.8)	34 (46.6)	30 (35.7)	0.167
	Yes	93 (59.2)	39 (53.4)	54 (64.3)	
**Pharmacotherapy profile**					
Number of prescribed medications	Median (IQR)	5 (3–7)	4 (3–6)	5 (4–8)	0.012
Polypharmacy	No	86 (54.8)	46 (63.0)	40 (47.6)	0.053
	Yes	71 (45.2)	27 (37.0)	44 (52.4)	
Hyperpolypharmacy	No	143 (91.1)	69 (94.5)	74 (88.1)	0.159
	Yes	14 (8.9)	4 (5.5)	10 (11.9)	

Note. Values are presented as *n* (%) unless otherwise specified. *p*-values were calculated using chi-square tests or Mann–Whitney U tests, as appropriate. Polypharmacy was defined as the prescription of five or more medications; hyperpolypharmacy was defined as the prescription of ten or more medications. IQR, interquartile range.

**Table 2:** Prevalence of medication safety gaps according to care setting.

Outcome	Overall, *n/N* (%)	Outpatient Care, *n/N* (%)	Hospitalization, *n/N* (%)	*p*-Value
Beers-defined potentially inappropriate medication	73/157 (46.5)	40/73 (54.8)	33/84 (39.3)	0.052
STOPP-defined potentially inappropriate prescribing	78/157 (49.7)	43/73 (58.9)	35/84 (41.7)	0.031
START-defined potential prescribing omission	83/157 (52.9)	31/73 (42.5)	52/84 (61.9)	0.015
Overall medication safety gaps	124/157 (79.0)	56/73 (76.7)	68/84 (81.0)	0.515

Note. *p*-values were calculated using Pearson’s chi-square test. The composite outcome of overall medication safety gaps was defined as the presence of at least one Beers-defined potentially inappropriate medication, STOPP-defined potentially inappropriate prescribing, or START-defined potential prescribing omission. PIM, potentially inappropriate medication; STOPP, Screening Tool of Older Persons’ Prescriptions; START, Screening Tool to Alert to Right Treatment.

**Table 3:** Number of medication-related problems identified per participant according to care setting.

Outcome	Overall, Median (IQR), Range	Outpatient Care, Median (IQR), Range	Hospitalization, Median (IQR), Range	*p*-Value
Beers-defined potentially inappropriate medications	0 (0–1), 0–4	1 (0–2), 0–4	0 (0–1), 0–4	0.014
STOPP-defined potentially inappropriate prescribing problems	0 (0–2), 0–3	1 (0–2), 0–3	0 (0–1), 0–3	0.009
START-defined potential prescribing omissions	1 (0–2), 0–4	0 (0–1), 0–3	1 (0–2), 0–4	0.006

Note. Values are presented as median (interquartile range) and observed minimum–maximum range. Between-setting comparisons were performed using two-sided asymptotic Mann–Whitney U tests. IQR, interquartile range; STOPP, Screening Tool of Older Persons’ Prescriptions; START, Screening Tool to Alert to Right Treatment.

**Table 4:** Crude and adjusted prevalence ratios for medication safety gaps according to care setting.

Outcome	Care Setting	Crude PR (95% CI)	*p*-Value	Adjusted PR (95% CI)	*p*-Value
Beers-defined potentially inappropriate medication	Outpatient care	Reference	—	Reference	—
	Hospitalization	0.72 (0.52–1.00)	0.052	0.76 (0.56–1.02)	0.068
STOPP-defined potentially inappropriate prescribing	Outpatient care	Reference	—	Reference	—
	Hospitalization	0.71 (0.52–0.97)	0.031	0.74 (0.55–0.99)	0.043
START-defined potential prescribing omission	Outpatient care	Reference	—	Reference	—
	Hospitalization	1.46 (1.08–1.98)	0.015	1.39 (1.04–1.86)	0.026
Overall medication safety gaps	Outpatient care	Reference	—	Reference	—
	Hospitalization	1.06 (0.90–1.24)	0.515	1.02 (0.89–1.18)	0.759

Note. PR, prevalence ratio; CI, confidence interval; STOPP, Screening Tool of Older Persons’ Prescriptions; START, Screening Tool to Alert to Right Treatment. Crude and adjusted PRs were estimated using Poisson regression with robust variance. Adjusted models included age group, sex, polypharmacy, and multimorbidity. The reference category was outpatient care.

**Table 5:** Factors associated with overall medication safety gaps.

Variable	Category	Overall Medication Safety Gaps, *n/N* (%)	Crude PR (95% CI)	*p*-Value	Adjusted PR (95% CI)	*p*-Value
Care setting	Outpatient care	56/73 (76.7)	Reference	—	Reference	—
	Hospitalization	68/84 (81.0)	1.06 (0.90–1.24)	0.515	1.02 (0.89–1.18)	0.759
Sex	Male	63/82 (76.8)	Reference	—	Reference	—
	Female	61/75 (81.3)	1.06 (0.90–1.24)	0.491	1.04 (0.90–1.20)	0.603
Age group	60–69 years	47/66 (71.2)	Reference	—	Reference	—
	70–79 years	45/55 (81.8)	1.15 (0.95–1.40)	0.153	1.09 (0.91–1.31)	0.340
	≥80 years	32/36 (88.9)	1.25 (1.03–1.51)	0.023	1.16 (0.98–1.37)	0.084
Polypharmacy	No	61/86 (70.9)	Reference	—	Reference	—
	Yes	63/71 (88.7)	1.25 (1.08–1.45)	0.003	1.19 (1.04–1.36)	0.011
Multimorbidity	No	44/64 (68.8)	Reference	—	Reference	—
	Yes	80/93 (86.0)	1.25 (1.06–1.48)	0.008	1.17 (1.01–1.35)	0.036
Chronic kidney disease	No	98/129 (76.0)	Reference	—	Reference	—
	Yes	26/28 (92.9)	1.22 (1.06–1.41)	0.006	1.12 (0.99–1.27)	0.071

Note. PR, prevalence ratio; CI, confidence interval. Crude and adjusted PRs were estimated using Poisson regression with robust variance. The adjusted model included care setting, sex, age group, polypharmacy, multimorbidity, and chronic kidney disease. The composite outcome of overall medication safety gaps was defined as the presence of at least one Beers-defined PIM, STOPP-defined PIP, or START-defined PPO.

## Data Availability

The de-identified data supporting the findings of this study and the SPSS syntax used for data management and statistical analysis are available from the corresponding author upon reasonable request, subject to the institutional and ethical conditions governing access to clinical-record data.

## Supplementary Materials

The following supporting information can be downloaded at: https://www.mdpi.com/article/10.3390/healthcare14152314/s1, Table S1: Most frequent inappropriate pharmacotherapy criteria identified; Table S2: Sensitivity analysis using hyperpolypharmacy instead of polypharmacy in the adjusted modified Poisson regression models.

## Abbreviations

The following abbreviations are used in this manuscript:

AGS	American Geriatrics Society
CI	Confidence interval
IQR	Interquartile range
PIM	Potentially inappropriate medication
PIP	Potentially inappropriate prescribing
PPO	Potential prescribing omission
PR	Prevalence ratio
SPSS	Statistical Package for the Social Sciences
START	Screening Tool to Alert to Right Treatment
STOPP	Screening Tool of Older Persons’ Prescriptions
