# Supplementary material for: Medication Safety Gaps in Older Adults Across Outpatient and Inpatient Care in Peru: Evidence from Beers and STOPP/START Criteria
Source: Healthcare (Basel). 2026 Jul 31;14(15):2314. doi: 10.3390/healthcare14152314 (PMC13465604; doi:10.3390/healthcare14152314)
Supplement: Supplementary file 1 [file healthcare-14-02314-s001.zip › healthcare-4399329-supplementary.pdf]

Supplementary Table S1. Most frequent inappropriate pharmacotherapy criteria identified

Tool	Criterion or medication-related problem	Overall, n (%)	Outpatient care, n (%)	Hospitalization, n (%)
**Beers**	Long-term use of nonsteroidal anti-inflammatory drugs in older adults at gastrointestinal or renal risk	24 (15.3)	16 (21.9)	8 (9.5)
**Beers**	Benzodiazepines or Z-drugs in older adults because of risk of falls, sedation, or cognitive impairment	21 (13.4)	14 (19.2)	7 (8.3)
**Beers**	Proton pump inhibitors without clear long-term indication	19 (12.1)	13 (17.8)	6 (7.1)
**Beers**	Anticholinergic medications in patients with cognitive or fall risk	14 (8.9)	9 (12.3)	5 (6.0)
**Beers**	Sulfonylureas with higher hypoglycemia risk in older adults	11 (7.0)	6 (8.2)	5 (6.0)
**STOPP**	Drug prescribed beyond the recommended duration without documented reassessment	29 (18.5)	20 (27.4)	9 (10.7)
**STOPP**	Therapeutic duplication within the same pharmacological class	17 (10.8)	10 (13.7)	7 (8.3)
**STOPP**	Medication increasing fall risk in a patient with previous fall or frailty markers	15 (9.6)	8 (11.0)	7 (8.3)
**STOPP**	Nonsteroidal anti-inflammatory drug use in high-risk gastrointestinal, renal, or cardiovascular conditions	14 (8.9)	9 (12.3)	5 (6.0)
**START**	Omission of statin therapy in patients with diabetes or established cardiovascular disease when indicated	24 (15.3)	8 (11.0)	16 (19.0)
**START**	Omission of antiplatelet therapy in patients with documented atherosclerotic cardiovascular disease when indicated	19 (12.1)	6 (8.2)	13 (15.5)
**START**	Omission of renin–angiotensin system blockade in diabetes, hypertension, heart failure, or proteinuric kidney disease when indicated	16 (10.2)	5 (6.8)	11 (13.1)
**START**	Omission of calcium/vitamin D or antiresorptive therapy in patients with osteoporosis or high fracture risk when indicated	14 (8.9)	5 (6.8)	9 (10.7)
**START**	Omission of inhaled maintenance therapy in chronic obstructive pulmonary disease or asthma when clinically indicated	10 (6.4)	4 (5.5)	6 (7.1)

Note. The table presents the most frequent medication-related problems identified using the Beers, STOPP, and START criteria. Percentages were calculated using the total number of participants within each care setting as the denominator.

Supplementary Table S2. Sensitivity analysis using hyperpolypharmacy instead of polypharmacy in the adjusted modified Poisson regression models.

Outcome	Hospitalization vs. outpatient care, adjusted PR (95% CI)	p-value	Hyperpolypharmacy vs. no hyperpolypharmacy, adjusted PR (95% CI)	p-value
**Beers-defined potentially inappropriate medication**	0.69 (0.49–0.98)	0.036	0.69 (0.30–1.58)	0.382
**STOPP-defined potentially inappropriate prescribing**	0.64 (0.46–0.88)	0.007	1.62 (1.07–2.44)	0.022
**START-defined potential prescribing omission**	1.40 (1.01–1.94)	0.046	1.36 (0.96–1.92)	0.080
**Overall medication safety gaps**	0.96 (0.82–1.13)	0.628	1.20 (0.96–1.50)	0.104

Note. PR, prevalence ratio; CI, confidence interval. Hyperpolypharmacy was defined as the prescription of ten or more medications. Models for the three criterion-specific outcomes were adjusted for age group, sex, hyperpolypharmacy, and multimorbidity. The model for overall medication safety gaps additionally included chronic kidney disease. Outpatient care and absence of hyperpolypharmacy were the reference categories.
